# First Description of the Clinical Activity of Avapritinib in Sporadic Mesenteric Desmoid Tumor

**DOI:** 10.1155/2024/8684418

**Published:** 2024-08-05

**Authors:** Rebecca Ganzon, Wei Chen, Gabriel Tinoco

**Affiliations:** ^1^ Division of Medical Oncology The Ohio State University Comprehensive Cancer Center-Arthur G. James Cancer Hospital and Richard J. Solove Research Institute, Columbus, Ohio, USA; ^2^ Department of Pathology The Ohio State University Comprehensive Cancer Center-Arthur G. James Cancer Hospital and Richard J. Solove Research Institute, Columbus, Ohio, USA

**Keywords:** avapritinib, desmoid tumor, GIST

## Abstract

Desmoid tumors (DTs) are rare and locally aggressive with a high rate of local recurrence even with optimal surgical resection. Systemic treatments are often utilized for desmoid cases with high risk of surgical morbidity or for local and symptomatic control of recurrent disease. However, the systemic treatment options for DTs are limited with limited responses. Avapritinib is a tyrosine kinase inhibitor (TKI) approved in 2020 for adults with unresectable or metastatic gastrointestinal (GI) stromal tumors (GISTs) harboring a platelet-derived growth factor receptor alpha (PDGFRA) Exon 18 mutation, including D842V mutations. In this case report, we describe a 55-year-old man with a history of D842V-mutant gastric GIST who presented several years after complete resection of the GIST with an enlarging soft tissue mass in the small intestine. After a nondiagnostic biopsy, the patient was started on avapritinib due to concerns for recurrent D842V-mutant GIST. The tumor had a partial response to treatment by RECIST 1.1 criteria, and the patient underwent surgical resection. The final pathology report revealed a sporadic DT. To our knowledge, this is the first known description of the activity of avapritinib in the treatment of a sporadic mesenteric DT, which is relevant given the limited treatment options for patients with this diagnosis. This clinical finding may be worth exploring in a dedicated clinical trial.

## 1. Introduction

Desmoid tumor (DT, also known as desmoid fibromatosis) is a rare, locally aggressive tumor of the fibroblastic connective tissue with an annual incidence of 2–4 per 1 million people annually [1]. DT arises in the deep tissue and tends to be locally aggressive and infiltrative in nature. While desmoids have a proclivity for local recurrence and sometimes are multifocal within one region, they are nonmalignant as they are unable to metastasize. An expert soft tissue pathologist should confirm diagnosis of DT; molecular analysis is highly recommended as DT is grossly characterized into one of two mutually exclusive subtypes: sporadic DT, which harbors a somatic B-catenin mutation (e.g., CTNNB1), and those that arise in the context of familial adenomatous polyposis and harbor a germline APC mutation (about 10% of cases) [2]. The treatment of DT is often challenging due to its inherent biological unpredictability and variable location, and it is recommended that these patients seek access to experienced multidisciplinary teams to tailor an optimal treatment plan [3]. For resectable tumors, observation and surgical resection are the most common treatment modalities. However, for patients with large tumors causing morbidity, pain, and functional limitations, or for patients with unresectable tumors, systemic options are an important part of disease management; however, these systemic treatments are extremely limited (see Table 1).

Gastrointestinal (GI) stromal tumor (GIST) is a rare soft tissue malignancy with an annual incidence of 15 per 1 million people annually. It is most commonly diagnosed in individuals over the age of 50, with equal incidence among males and females [11, 12]. It is the most prevalent soft tissue sarcoma (STS) of the GI tract and the fourth most common STS in general. Although GIST can occur anywhere along the GI tract, it most commonly affects the stomach (60%) or small intestine (30%) [11, 12]. The most common primary activating mutations in GIST come from alterations in tyrosine kinase genes, most frequently KIT or platelet-derived growth factor receptor alpha (PDGFRA). The majority of GIST cases harbor a mutation in KIT Exon 11 (60%–70%) or KIT Exon 9 (10%). PDGFRA mutations are less common (up to 10%), with the most frequent mutation occurring at PDGRA Exon 18 D842V (5%) [13, 14]. Around 10% of cases do not harbor a KIT or PDGFRA mutation and have historically been referred to as “wild type.” A significant number (around 40% of GIST cases that lack KIT or PDGFRA mutations) have loss-of-function mutations in the succinate dehydrogenase (SDH) subunits or loss of SDH complex iron sulfur subunit B (SDHB) protein expression and are referred to as SDH-deficient GIST [15]. Primary mutations in BRAF, NF1, or other genes are less commonly seen [16]. There are currently no US Food and Drug Administration (FDA)–approved treatments for these wild-type GIST mutations; historically, these can be much more challenging to treat if the disease is advanced.

Until more recently, surgical resection was the standard primary treatment modality for both DT and GIST, but recurrence rates are still around 40% in DT [17, 18] and up to 50% for GIST [19, 20]. However, with the advent of tyrosine kinase inhibitors (TKIs), there has been an increasing role for systemic therapies in the management of these tumors. The approved treatment regimens for DT and GIST are listed in Tables 1 and 2. In 2020, avapritinib was approved as a first-line treatment for GIST tumors with confirmed PDGFRA D842V mutation GIST, a subtype of GIST that is generally resistant to standard GIST treatment regimens.

## 2. Case Presentation

**Figure 1:**
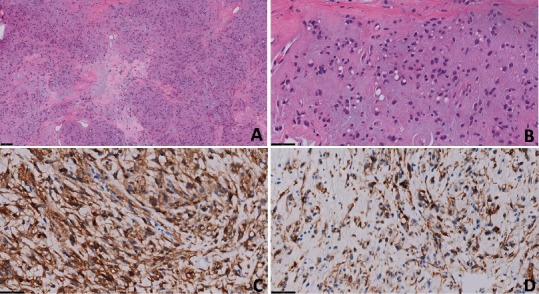
(A–D) Histomorphology of perigastric epithelioid GIST. (A, B) Low and higher power showing epithelioid tumor cells with focal perinuclear vacuoles, hematoxylin and eosin (H&E) stain. (C, D) DOG-1 and CD117/c-kit immunohistochemical stains are diffusely positive, supporting the diagnosis of GIST. Scale bars: (A) 100 *μ*m; (B–D) 50 *μ*m.

**Figure 2:**
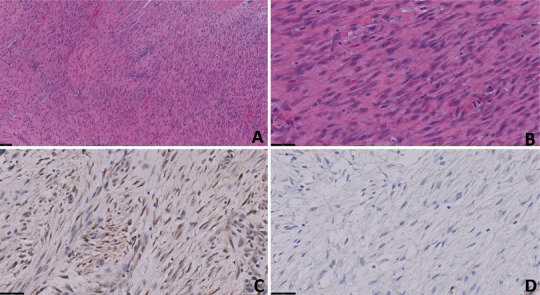
(A–D) Histomorphology of small bowel mesenteric desmoid-type fibromatosis. (A, B) Low and higher power showing fascicles of spindle cells with small nucleoli, H&E stain. (C) Beta-catenin immunohistochemical stain shows nuclear positivity, supporting the diagnosis of mesenteric desmoid-type fibromatosis. (D) DOG-1 immunohistochemical stain is negative. Scale bars: (A) 100 *μ*m; (B–D) 50 *μ*m.

A 55-year-old man presented to the emergency room with acute, band-like abdominal pain. A CT scan of his abdomen and pelvis demonstrated hemoperitoneum and a 5.0 × 3.5 cm mass in the lesser gastric fundus. A fine needle aspiration (FNA) was performed via endoscopic ultrasound (EUS), and final pathology reported GIST (Figures 1 and 2). Due to the acute presentation, neoadjuvant treatment was not recommended; the patient underwent laparoscopic partial gastrectomy. The largest fragment of the tumor resected measured 6.2 cm with a reported mitotic rate of 4 per 20 high-power fields (HPFs). Given the patient's high-risk presentation with a spontaneous hemoperitoneum from tumor rupture, which could lead to contamination of the peritoneal cavity, medical oncology recommended adjuvant treatment. The patient started daily imatinib at 400 mg by mouth while next-generation testing of the tumor was in process to determine the patient's specific primary GIST mutation.

**Figure 3:**
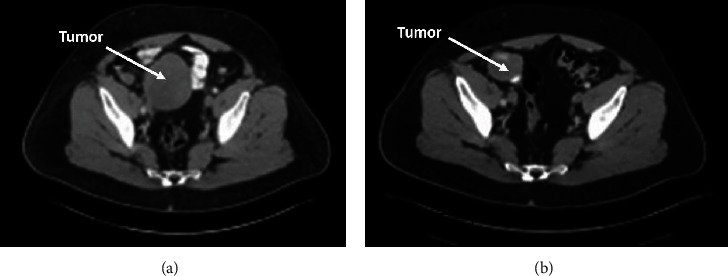
Response to treatment. Imaging showing response to treatment from (a) June 2021 (baseline) to (b) June 2022 (after 9 months on avapritinib).

Mutation analysis came back reporting an Exon 18 D842V mutation, which has been linked to primary resistance to imatinib [32–34]. Given this mutation, the patient was instructed to discontinue imatinib. He was then followed with CT scans, per National Comprehensive Cancer Network (NCCN) guidelines [16], for 23 months, with no evidence of recurrent or metastatic disease; however, he became lost to follow-up for 20 months. When he re-established care (around 43 months from initial gastric D842V GIST diagnosis), restaging scans showed a new 3.3 × 3.7 cm distal ileal soft tissue mass as well as three small, subcentimeter lesions, concerning for peritoneal implants near the stomach and anterior omentum. Due to COVID-19-related concerns, the patient delayed his medical treatment for an additional 9 months, at which time repeat imaging showed that the ileal mass had enlarged to 8.5 × 6.9 cm, whereas the other areas appeared stable. An endoscopic biopsy of the ileal mass was nondiagnostic due to the very scant amount of tissue collected; a repeated biopsy was felt to be technically challenging, and the patient refused an open surgery. In consultation with surgical oncology and given the previous medical history of D842V-mutant GIST with high-risk features with no ability to complete an effective adjuvant chemotherapeutic regimen, the decision was made to start neoadjuvant treatment with avapritinib daily at 300 mg by mouth. (Avapritinib was approved by the FDA as first-line therapy for patients with PDGFRA Exon 18 mutation, including D842V mutation, in January 2020, and was not approved or available at time of his initial GIST diagnosis.) The patient required dose reductions due to Grade 2 cognitive impairment, anasarca, and fatigue, eventually tolerating (from a toxicity standpoint) 50 mg by mouth daily every other week. He underwent imaging every 3 months while on treatment, achieving partial radiological response to treatment. Imaging after 9 months (Figure 3) showed that the ileal mass had significantly decreased to 3.7 × 4.5 cm; the patient underwent resection at that time.

Avapritinib was discontinued 5 days prior to surgery. Surprisingly, the final pathology reported a 6.4 cm DT with next-generation sequencing confirming a CTNNB1 p.T41A hotspot mutation, supporting the diagnosis of desmoid-type fibromatosis. The three small, subcentimeter lesions were surgically excised, and the final pathology report confirmed that they were benign fibroadipose tissue. The patient is now on surveillance with no evidence of recurrent or metastatic disease from either the GIST or DTs. Avapritinib was not restarted postoperatively, and his side effects fully resolved and returned to baseline.

## 3. Discussion

To our knowledge, this is the first case describing the clinical activity of avapritinib in DTs. It also addresses two very rare conditions: spontaneous mesenteric desmoid fibromatosis and D842-mutant GIST. Treatment options for these tumor types have some overlap as they are both commonly treated with TKIs, albeit with significantly different responses. There has been notable progress in GIST treatment in recent years with the approval of two new treatments in 2020, including the FDA approval of avapritinib, which has opened a new treatment option for patients with Exon 18 D842V-mutant GIST who historically had poorer prognosis due to primary resistance to imatinib and other TKIs [32–34].

The precise biological mechanisms underlying the efficacy of TKIs in the treatment of DTs have yet to be comprehensively elucidated, as often this family of drugs targets multiple tyrosine kinases, thereby affecting multiple pathways [35].

Avapritinib selectively targets PDGRA, PDGFRB, KIT, and CSFR1 [36], which correspondingly serve as targets for other TKIs with clinical activity in the treatment of DTs. Consequently, it is plausible that avapritinib demonstrates analogous therapeutic activity to other TKIs in this context.

Despite the publication of a case series [37], a definite association between GIST tumors and desmoids has not been established. Trauma is a known risk factor for the development of sporadic DTs [38, 39]. In this case, previous surgical intervention may have contributed to the development of the DT.

Despite recent progress in GIST treatment, the treatment of PDGFRA Exon 18 D842V-mutant tumors remains challenging, mainly due to side effects associated with avapritinib.

DTs have limited treatment options, and there have not been breakthroughs since sorafenib or recently approved nirogacestat. This unique case demonstrates a significant response to treatment with the use of avapritinib in a DT, which was unexpected and previously undocumented. Based on this case study, it seems that avapritinib may have some use in the treatment of DTs, though this has never been formally investigated; this poses an opportunity for further research.

## Figures and Tables

**Table 1:** GIST treatments.

**Drug**	**Treatment class**	**ORR (CR + PR) (%)**	**Median PFS (months)**	**Administration**
Imatinib [4, 5]	TKI	53.7	18.9	PO, daily
Sunitinib [6]	TKI	7	5.6	PO, daily
Regorafenib [7]	TKI	4.5	4.8	PO, daily
Ripretinib [8]	TKI	9.4 at initial cutoff 11.8 in long-term follow-up analysis	6.3	PO, daily
Avapritinib [9, 10]	TKI	88 (in D842V mutation) vs. 21 (in non-D842V mutant)	4.2 (in non-D842V mutant)	PO, daily

Abbreviations: CR, complete response; ORR, objective response rate; PFS, progression-free survival; PO, by mouth; PR, partial response; TKI, tyrosine kinase inhibitor.

**Table 2:** DT treatments.

**Drug**	**Treatment class**	**ORR (CR + PR) (%)**	**12-month PFS (%)**	**Administration**
Imatinib [21–23]	TKI	6–19	59–66	PO, daily
Sorafenib [24]	TKI	33	89	PO, daily
Pazopanib [25]	TKI	37	86	PO, daily
MTX + VBN [25–27]	Chemotherapy	19–40	58–79	IV, weekly
Doxil [28, 29]	Chemotherapy	36	75 at median 14 months	IV, every 4 weeks
Doxorubicin + dacarbazine [30]	Chemotherapy	66	Not reported	IV, every 4 weeks
Nirogacestat [31]	*γ*-Secretase inhibitor	41	85	PO, twice daily

Abbreviations: CR, complete response; IV, intravenous; ORR, objective response rate; PFS, progression-free survival; PO, by mouth; PR, partial response; TKI, tyrosine kinase inhibitor.

## Data Availability

All data underlying the results are available as part of the manuscript, and no additional source data are required.
